# How reduction temperature influences the structure of perovskite-oxide catalysts during the dry reforming of methane†

**DOI:** 10.1039/d4su00483c

**Published:** 2024-10-10

**Authors:** Florian Schrenk, Lorenz Lindenthal, Hedda Drexler, Tobias Berger, Raffael Rameshan, Thomas Ruh, Karin Föttinger, Christoph Rameshan

**Affiliations:** a Chair of Physical Chemistry, Montanuniversität Leoben Austria christoph.rameshan@unileoben.ac.at; b Institute of Materials Chemistry, Technische Universität Wien Austria

## Abstract

Dry reforming of methane is a promising reaction to convert CO_2_ and combat climate change. However, the reaction is still not feasible in large-scale industrial applications. The thermodynamic need for high temperatures and the potential of carbon deposition leads to high requirements for potential catalyst materials. As shown in previous publications, the Ni-doped perovskite-oxide Nd_0.6_Ca_0.4_Fe_0.97_Ni_0.03_O_3_ is a potential candidate as it can exsolve highly active Ni nanoparticles on its surface. This study focused on controlling the particle size by varying the reduction temperature. We found the optimal temperature that allows the Ni nanoparticles to exsolve while not yet enabling the formation of deactivating CaCO_3_. Furthermore, the exsolution process and the behaviour of the phases during the dry reforming of methane were investigated using *in situ* XRD measurements at the DESY beamline P02.1 at PETRA III in Hamburg. They revealed that the formed deactivated phases would, at high temperatures, form a brownmillerite phase, thus hinting at a potential self-healing mechanism of these materials.

## Abstract 2

Effects of pre-treatment temperatures on the catalytic activity of a Ni-doped perovskite for dry reforming of methane were studied. Particles form at optimal temperatures that are stable under reaction conditions, shown by *in situ* XRD measurements.

## Graphical abstract

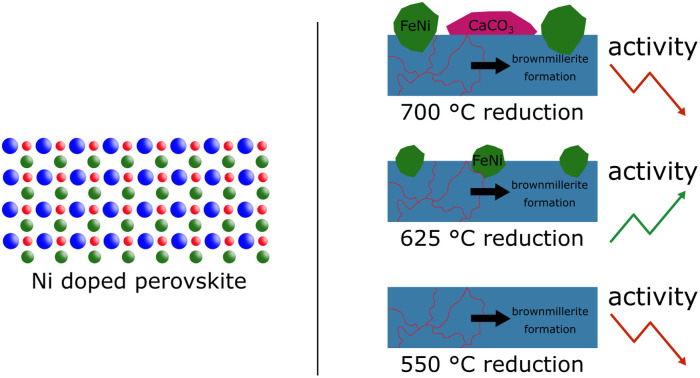

**Sustainability spotlight**

One possible way to reach the goal of carbon-neutrality in industry is the conversion of greenhouse gases into value-added chemicals. Processes that achieve this usually require catalysts, thus a fundamental understanding is paramount for the development of such catalytic systems. In this study, we investigate materials that show great potential for methane dry reforming, keeping in line with the UN Sustainability Development Goal 13 (Climate Action). Moreover, our materials mainly consist of fairly cheap and abundant materials, such as iron and calcium, while keeping the concentrations of the catalytically active metal – nickel – low. While neodymium is a critical raw element as well, further research to replace it with a non-critical resource is already underway. Therefore, our study also contributes to SDG 7 (Affordable and Clean Energy).

## 1 Introduction

The catalytic conversion of methane (CH_4_) and carbon dioxide (CO_2_) to synthesis gas (H_2_ + CO), known as dry reforming of methane (DRM), could play a role in slowing down climate change and transitioning into a sustainable energy future.^1^ Even though the demanding reaction conditions do not allow an economically viable or even CO_2_-neutral process with current technologies, the two reactants belong to the most critical greenhouse gases and are produced on a large-scale in biomass conversion processes.^2^ The product mixture is one of the fundamental building blocks of the modern chemical industry.^3^ The Fischer–Tropsch reaction (*e.g.*, production of hydrocarbons) and methanol production are two potential applications of synthesis gas produced from DRM. The lower H_2_/CO ratio produced influences the Fischer–Tropsch reaction's selectivity.^4^ The overall reaction is displayed in eqn (1):

1 CH_4(g)_ + CO_2(g)_ ⇌ 2H_2(g)_ + 2CO_(g)_** **(Δ*H*^298^_r_ = +247 kJ mol^−1^)

The reaction is thermodynamically most favourable in atmospheric pressure.^1^ However, the reaction is endothermic, requiring high reaction temperatures of up to 1000 °C for conversions relevant to industrial applications.^5^ These high reaction temperatures pose a significant challenge as they lead to the deactivation of most catalysts *via* the sintering of active nanoparticles and coke formation. In contrast to other methane reforming processes, such as autothermal reforming and steam reforming, DRM produces CO-rich product mixtures, which can benefit downstream processes. This complexity and the potential benefits have sparked a high interest in developing an effective catalyst that is stable under the extreme conditions necessary for industrial applications. Aside from the stability, one must keep the tendency for side reactions in mind. DRM is especially prone to facilitating reverse water-gas shift (rWGS) as an unwanted reaction (eqn (2)).^1^ In rWGS, H_2_ and CO_2_ react to form CO and H_2_O, leading to a shift in the product ratio of DRM to a CO-rich atmosphere. As discussed, the CO-rich product is actually an advantage of DRM. However, there are limits to its usability, especially if it should be used directly for the Fischer–Tropsch reaction and without further dilution in hydrogen after rWGS,^5^ as the H_2_/CO ratio is crucial for Fischer–Tropsch selectivity. Another important side reaction is the Boudouard equilibrium (eqn (3)), which shifts to the product side as temperature increases, making applications of DRM feasible only at temperatures above 750 °C to avoid coking.^1^ A third important side reaction is methane pyrolysis, where methane decomposes directly on a metallic catalyst, forming hydrogen and solid carbon (eqn (4)).

2 H_2(g)_ + CO_2(g)_ ⇌ H_2_O_(g)_ + CO_(g)_** **(Δ*H*^298^_r_ = +42.1 kJ mol^−1^)

3 C_(s)_ + CO_2(g)_ ⇌ 2CO_(g)_** **(Δ*H*^298^_r_ = +172.5 kJ mol^−1^)

4 CH_4(g)_ ⇌ 2H_2(g)_ + 4C_(s)_** **(Δ*H*^298^_r_ = +74.9 kJ mol^−1^)

In literature, two main approaches are used for DRM catalysts: utilisation of transition metal-supported catalysts (mostly Ni is chosen)^6,7^ or catalysts containing noble metal elements.^8,9^ The second group of materials is less prone to coking, but their expensive components make them less interesting for large-scale industrial applications. The first group uses more widely available transition metals but suffers from the formation of carbon nanotubes or carbon deposits on their surface.^10^

Perovskite-type oxides (further referred to as perovskite-oxides) have attracted attention, especially in the areas of heterogeneous catalysis and solid-state ionics.^11–13^ A property that makes perovskite-oxides attractive for heterogeneous catalysis is their ability to form metallic nanoparticles when treated in reducing conditions.^14^ This process, known as exsolution, produces not only highly distributed nanoparticles, but they are also socketed into the surface, making them resistant to sintering as their surface mobility is dramatically decreased.^15,16^ This leads to incredible long-term stability compared to materials consisting of deposited nanoparticles on an oxide surface. In a recent study, Kim *et al.* showed that the exsolved nanoparticles grow slightly if the material is exposed to reducing conditions for up to 24 h, and they also change their shape as they facet.^16^ However, in long-term experiments, these faceted nanoparticles showed a high conversion of over 94% for 390 h for DRM, while reference systems showed an apparent decrease in activity. In contrast to conventional nanoparticles used for DRM, particles produced *via* exsolution are bigger than their counterparts^16^ with average sizes up to 50 nm. Even though the exsolution phenomenon is well studied, a lot of questions are still open: from the basic mechanism as described by Gao *et al.*^17^ and Wang *et al.*^18^ to how the particle density can be influenced^19^ to how the basic makeup of the material influences its catalytic activity.^20^ The authors want to recommend the recently published roadmap paper on this topic for a more detailed insight on the challenges and open questions regarding perovskite-oxide materials.^21^

In a previous study, the applicability of perovskite-oxides for DRM^22^ was shown, comparing Ni- and Co-doped systems in contrast to a Fe-based backbone of the material. The term “Fe-based backbone” refers to the material without any doping on the B-site either due to not doping it in the first place or exsolving the dopant. The main findings of this work were that the Ni-doped system outperformed the Co-doped one. The deposited carbon species vary with temperature and play a vital role in the reaction mechanism as carbonate intermediates form. Therefore, we proposed a temperature-dependent switch between a “DRM” and a “reverse water-gas shift pathway”. The main difference is that in the second pathway (at lower temperatures), the formed and still adsorbed hydrogen spills over to the perovskite support, leading to the formation water. This process has already been shown in other works.^23^ At higher temperatures, the atomic hydrogen reacts on the metallic nanoparticle with another hydrogen, combining to H_2_ that desorbs, leading to “true” DRM.

Following up on previous work,^22^ this paper focuses on the effect of the temperature of the reductive pre-treatment on the catalytic activity. Therefore, we compared the catalyst from our previous work,^22^ Nd_0.6_Ca_0.4_Fe_0.97_Ni_0.03_O_3−*δ*_ (NCF-Ni), with its B-site undoped version Nd_0.6_Ca_0.4_FeO_3−*δ*_ (NCF) under three different reduction temperatures: one chosen not to allow the formation of metallic nanoparticles, one where the exsolution sets in, and one where the exsolution is more pronounced but the backbone structure is already at the stability limit. In addition to the lab-based reactor and X-ray diffraction (XRD) measurements, a big part of the study utilised synchrotron-based *in situ* XRD measurements that allowed a time resolution in the seconds range, leading to insights into the exsolution process and its effect on the catalytic activity for DRM.

Considering the sustainability impact of the work, the fundamental character of this manuscript should be stressed. Dry reforming methane is interesting for sustainable production of chemicals, for reasons discussed above. This work investigates the prospects of a material class that could be used for this reaction. The concentration of the catalytically active metal – Ni – could already be reduced to 0.6 mol% in total. The A-site of the material still contains Nd, a critical raw material. However, this study mainly focuses on the B-site of the material and does not claim to provide a complete solution for DRM. Further research, especially on the effect of the A-site, is therefore still necessary.

## 2 Experimental

### 2.1. Preparation of the materials

The catalysts were synthesised following a modified Pechini approach described in more detail in previous work.^20,24^ The respective amounts of the starting materials Nd_2_O_3_ (99.9%, Strategic Elements), CaCO_3_ (99.95%, Sigma-Aldrich), Fe (99.5%, Sigma-Aldrich), and Ni(NO_3_)_3_·6H_2_O (98%, Alfa Aesar) were dissolved in HNO_3_ (65% Merck) or H_2_O and mixed. Citric acid (99.9998% trace metal pure, Fluka) was added as a complexing agent, and the mixture was heated until gel formation and self-ignition. The formed powders were calcined for three hours at 800 °C in air. After grinding of the products, they were analysed with powder XRD for phase purity and with BET for surface area. The diffractograms of the pristine samples and the surface areas can be found in the ESI (Fig. S1 and Table S1).†

Powder XRD measurements were taken on a PANalytical X'Pert Pro diffractometer (Malvern Panalytical, Malvern, UK) in Bragg–Brentano geometry using a mirror for separating the Cu Kα_1,2_ radiation and an X'Celerator linear detector (Malvern Panalytical, Malvern, UK). SEM images were recorded using a Quanta 250 FEGSEM (FEI Company) microscope in the USTEM facilities of TU Wien.

### 2.2. Pre-treatments and catalytic tests

The materials were subjected to reducing pre-treatments and catalytic tests using a custom-built reactor system, which has already been used in previous work.^20,22^ In short, it consists of a gas-mixing unit built from steel tubes (Burde & Co, Vienna, Austria) and fittings (Swagelok, Solon, USA) equipped with an optional saturator for humid gas environments. The catalytic materials were placed inside a quartz tube (6 mm outer diameter, 4 mm inner diameter) on top of quartz wool inside an oven controlled by a K-type thermocouple inside the catalyst bed. Off-gas analysis was performed with a Micro-Gas-Chromatograph (Micro-GC, Fusion 3000 A, Inficon), collecting data points every two to three minutes.

The samples were mounted inside the reactor and heated under Ar atmosphere to 600 °C. Afterwards, the gas was switched to pure O_2_ for 30 minutes (flow: 10 mL min^−1^). Then, the gas atmosphere was switched to Ar again to purge the oxygen, and the temperature was changed to the respective temperature of the reductive pre-treatment (550 °C, 625 °C, and 700 °C, respectively). The atmosphere was changed to H_2_/H_2_O by running 10 mL per min H_2_ through a water saturator. This leads to an H_2_/H_2_O ratio of roughly 32 : 1. The reduction period was set to one hour. Afterwards, the atmosphere was again changed to Ar. The reactor was cooled down to the respective temperature of the subsequent experiments (room temperature for the reduction experiments or 350 °C for further catalytic tests). At this temperature, the gas atmosphere was changed to DRM atmosphere (Ar : CH_4_ : CO_2_ = 3 : 2 : 1 with a total flow of 20 mL min^−1^). After stabilising the atmosphere, the temperature was raised by 5 °C min^−1^ up to 500 °C followed by 3 °C min^−1^ up to 950 °C.

### 2.3. Synchrotron XRD measurements

For the synchrotron experiments at DESY beamline P02.1 at PETRA III (Deutsches Elektronen Synchrotron, Hamburg, Germany), a lab-built flow cell (heated sample capillary connected to a gas flow system) was used. The samples were heated in Ar up to 600 °C before switching to an O_2_ atmosphere (10 mL min^−1^) for 30 minutes, mimicking the lab-based experiments. Afterwards, the samples were reduced in humidified hydrogen (10 mL min^−1^) for one hour at the respective temperature. The DRM reaction program (total gas flow 10 mL min^−1^, Ar : CH_4_ : CO_2_ = 3 : 2 : 1) was also replicated from the lab to achieve maximum comparability between the lab-based and synchrotron experiments. The XRD measurements were performed in transmission geometry with an energy of 60 keV and an area detector. The pictures were integrated to obtain 1D Data using the software DAWN.^25^ Data was analysed using GSAS II software^26^ and the ICDD PDF-4+ 2023 (ref. 27) database.

## 3 Results and discussion

### 3.1. Variation of the pre-treatment temperature

#### 3.1.1. Characterization after reduction

**Fig. 1:**
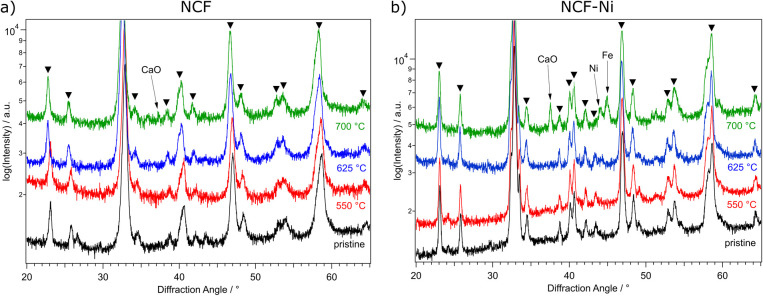
XRD measurements of NCF (a) and NCF-Ni (b) after one hour reduction in H_2_/H_2_O at 500 °C (red), 625 °C (blue), and 700 °C (green), respectively, as well as the comparison to the pristine catalyst (black). Reflexes marked with triangles can be assigned to the perovskite phase. NCF showed no additional phases after reduction at 550 °C and 625 °C. Only at a reduction temperature of 700 °C the formation of CaO is visible. For NCF-Ni, the post-reduction formation of Ni was already visible at 625 °C. After an increase in the reduction temperature to 700 °C, additional Fe and CaO formation could be observed.

**Fig. 2:**
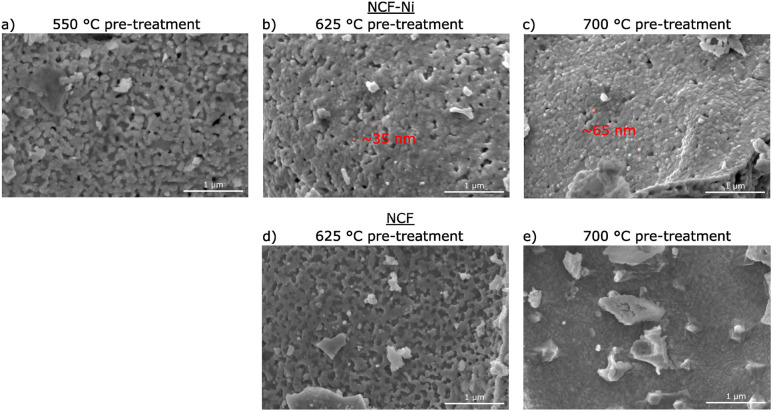
SEM images of NCF (d and e) and NCF-Ni (a–c) after a reductive pre-treatment at 550 °C (a), 625 °C (b and d) and 700 °C (c and e) respectively. For NCF-Ni, the size of the exsolved nanoparticles (red circles) increases with the rising temperature of the pre-treatment. For NCF, no exsolution is visible even after 700 °C pre-treatment.

As in previous work,^22^ NCF and NCF-Ni were reduced in a flow reactor for one hour. In this work, this reduction took place at various temperatures to characterise the activated state of the materials. After the reduction, the samples were returned to room temperature, and standard powder XRD (Fig. 1) and SEM (Fig. 2) measurements were performed.

In Fig. 1, the diffractograms of the reduced B-site undoped (hereinafter referred to simply as undoped) (Fig. 1a) and doped (Fig. 1b) catalysts are compared to a measurement of the pristine samples. Going from the bottom up, the untreated material is depicted, followed by the tracks for 550 °C (red), 625 °C (blue), and 700 °C (green) reduction temperature, respectively. Focusing on the undoped sample first, no changes are visible compared to the pristine sample. This is consistent with TPR measurements in previous work,^22^ which only exhibited formation of oxygen vacancies below these temperatures. However, when exposed to air, these vacancies would most likely fill up with oxygen again. So, it is unsurprising that no oxygen-deficient phases could be confirmed after this reduction step. This behaviour continues with the increase of the reduction temperature to 625 °C. Upon reaching 700 °C reduction temperature, CaO is formed, which is the product of A-site segregation of alkaline earth metals in perovskites, a well-known deactivation phenomenon.^28^

In Fig. 1b, the reduction of the Ni-doped sample is depicted. Again, the reduction at 550 °C did not lead to changes in the catalyst. Because of the above-mentioned reasons, oxygen vacancies formed at lower temperatures (as seen in ref. 22 for NCF-Ni) cannot be detected with *ex situ* XRD. Reflexes of both samples shifted to lower diffraction angles after the pre-treatment at 625 °C. This could indicate the formation of vacancies that are not re-filled in air. However, in contrast to the undoped sample, the formation of a metallic bcc phase (around 44.5°) could already be detected after the reduction at 625 °C. Due to the data quality achieved with this lab-based diffractometer, it is difficult to distinguish whether it is a pure Ni phase or a mixed Fe and Ni phase. After the reduction at 700 °C, two reflexes can be differentiated in the Fe/Ni area. The peak at 44.1° is likely a fcc Ni phase that is shifted to lower diffraction angles as Fe is incorporated into the crystal. The second reflex at 44.9° could be a bcc Fe phase with Ni incorporated, shifting it to higher diffraction angles. The exsolution of nanoparticles at this temperature is also in line with previous work.^22^ Increasing the reduction temperature further – to 700 °C – leads to the formation of CaO, a deactivating phase, as discussed before, and the brownmillerite phase present at lower temperatures is vanishing. Interestingly, the reduction temperature of 625 °C seems to yield an optimal phase composition as a metallic phase is exsolved from the host oxide while no CaO could be detected. Regarding efficiency, the reduction temperature of 550 °C for NCF was no longer investigated, as the change compared to 625 °C was negligible. The focus of this work lies on the more exciting catalysts NCF-Ni with its three states: before exsolution (550 °C reduction), optimal exsolution (625 °C reduction) and partially decomposed (700 °C reduction). Reference points at 625 °C and 700 °C will be the undoped counterpart.

Fig. 2 depicts SEM images for different reduction temperatures. Displayed in the top row is NCF-Ni reduced at 550 °C (Fig. 2a), 625 °C (Fig. 2b), and 700 °C (Fig. 2c). The undoped reference points are reduced at 625 °C (Fig. 2d) and 700 °C (Fig. 2e), shown in the bottom row. The scale bar on the bottom right of each picture is 1000 nm long. For NCF-Ni, the SEM images are consistent with the XRD measurements. The reduction at the lowest temperature leads to no discernible formations on the catalyst surface. Increasing the reduction temperature to 625 °C yields the formation of small spherical nanoparticles. Identical spherical particles could be observed after reduction at 700 °C. A couple of particles were measured (marked red in Fig. 2); this size analysis revealed that the particles grew from around 35 nm up to roughly 65 nm with increased temperature. It should be noted that no full-size analysis was performed, but the representative SEM images still demonstrate differences in the size of the nanoparticles after different reduction temperatures. NCF, however, does not show the formation of particles on its surface neither after 625 °C nor after 700 °C reduction temperature. After reduction at 700 °C, large particles with sharp edges became visible. These could correspond to the CaO formed. Looking at the underlying surface morphology, it becomes apparent that the porosity decreases with increasing reduction temperature. The level of this behaviour is surprising as the materials were already calcined at a higher temperature of 800 °C for three hours during synthesis. However, this calcination was performed in air. In contrast, the reduction was performed in humidified H_2_, which may alter the behaviour of the perovskite (*i.e.* formation of vacancies and increase of lattice mobility).^29,30^

#### 3.1.2. Catalytic activity

**Fig. 3:**
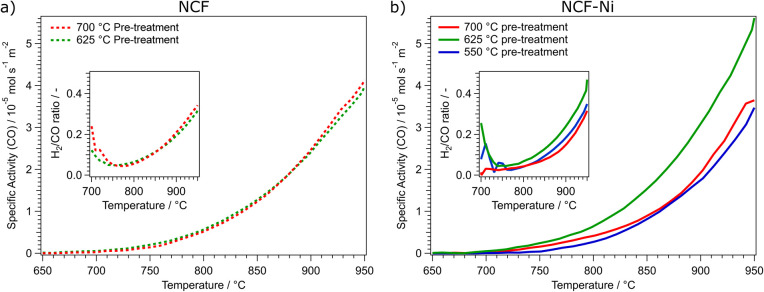
Comparison of the specific activity dependent on the reduction temperature of the pre-treatment for NCF ((a), left) and NCF-Ni ((b), right). NCF shows that an increase in the reduction temperature from 625 °C (green curve) to 700 °C (red curve) does not increase catalytic activity. For NCF-Ni, the increase of the reduction temperature from 550 °C (blue curve) to 625 °C (green curve) also increases the catalytic activity by roughly the same factor. However, a further increase of the reduction temperature to 700 °C (red curve) decreases the catalytic activity again by approximately the same factor. The inserts in each graph show the calculated H_2_/CO ratio during the temperature ramp. The nominal product ratio of 1 : 1 is not reached, as side reactions (*e.g.*, rWGS and Boudouard equilibrium) occur, as discussed in previous work.^1,22^

To measure the catalytic activity, the catalysts were subjected to a temperature ramp of 3 °C min^−1^ in a CH_4_ : CO_2_ : Ar = 2 : 1 : 3 atmosphere (called DRM atmosphere from here on out). The off-gases were analysed with a micro-GC. Fig. 3 depicts the calculated catalytic activity for the two materials and three reduction temperatures. The specific activity was calculated as the molar amount of CO produced normalized to the time (to include reactive gas flow) and surface area. Due to the nature of the perovskite-oxide type catalysts, it is not reasonable to calculate turnover frequencies as the active sites (*i.e.* size of nanoparticles and number of oxygen vacancies) change during reaction conditions.

As shown in Fig. 3a, the reduction temperature does not significantly influence the catalytic activity of the undoped material. Both cases produce substantial amounts of CO between 750 °C and 800 °C. The catalytic activity is continuously rising to 950 °C. The catalytic activity is remarkably similar over the whole temperature range. This is also reflected in the catalytic data presented in Table 1. Both undoped catalysts peak around 4.00 × 10^−5^ mol s^−1^ m^−2^ CO production. To quantify the catalytic activity during the temperature ramp, the parameters *T*_start_ and *T*_middle_ are listed. These values represent the temperatures at which the materials reached a catalytic activity of 5.00 × 10^−6^ mol s^−1^ m^−2^ and 2.50 × 10^−5^ mol s^−1^ m^−2^, respectively. In these figures, both undoped materials do not differ. Another critical factor during DRM is the ratio of H_2_ and CO produced. The ratio should be 1 as the same number of molecules are produced. However, due to several side reactions occurring (*e.g.*, rWGS or Boudouard equilibrium), the product ratio is shifted, generally to a CO excess;^1^ however, this is dependent on the temperature (at higher temperatures, the Boudouard reaction yields CO). In the insert in Fig. 3a, the H_2_/CO ratios of the undoped catalysts are depicted. Due to the low conversion rates at 700 °C, the product ratio has a high error. However, increasing the temperature and, therefore, the conversion means this value becomes more reliable. This may also explain the initial decrease in the ratio at temperatures below 750 °C. It could also be possible that the material is still being reduced at lower temperatures, leading to a CO excess. At this temperature, the H_2_/CO ratio reaches a minimum of around 0.05, meaning most of the produced hydrogen is converted, most likely to water, as the materials exhibit high activity for the rWGS reaction.^23^ The H_2_/CO ratio steadily increases to nearly 0.4 at 950 °C with increasing temperature. This means that around half of the theoretically produced hydrogen is converted in side reactions, even at the highest temperatures. At these temperatures, the Boudouard equilibrium is nearly entirely on the side of CO.

**Table 1:** Overview of the catalytic data of NCF and NCF-Ni for DRM after reduction at different temperatures. The material is denoted with the respective reduction temperature. The maximum specific activity for all measurements was recorded at the maximum temperature of 950 °C. To characterise the activity in more detail, the temperatures *T*_start_ and *T*_middle_ are reported. They describe the temperature at which the catalyst showed an activity of 5.00 × 10^−6^ mol s^−1^ m^−2^ (roughly 10% of the maximum specific activity of NCF-Ni 625 °C) and 2.50 × 10^−5^ mol s^−1^ m^−2^ (roughly 50% of the maximum specific activity of NCF-Ni 625 °C), respectively

Material	Max specific activity (CO)/mol s^−1^ m^−2^	*T*_start_/°C	*T*_middle_/°C
NCF-Ni 550	3.47 × 10^−5^	830 °C	925 °C
NCF-Ni 625	**5.62 × 10** ^**−5**^	**785 °C**	**885 °C**
NCF-Ni 700	3.85 × 10^−5^	810 °C	920 °C
NCF 625	4.00 × 10^−5^	800 °C	910 °C
NCF 700	4.14 × 10^−5^	800 °C	910 °C

In Fig. 3b, the catalytic activities for the Ni-doped catalysts are shown. It becomes clear that the medium reduction temperature of 625 °C is the most advantageous for the catalytic reaction. Coincidentally, too low (550 °C) and too high (700 °C) reduction temperatures lead to a similar catalytic activity. The main difference between these two measurements is that the reduction at 700 °C gave rise to CO production already at lower temperatures. Specifically, in a temperature range between 700 °C and 775 °C the catalytic activity of NCF-Ni-700 is significantly higher than that of NCF-Ni-550. However, increasing the temperature further, the catalytic activities of both materials become more similar. In contrast, the activity of NCF-Ni-625 is higher than the other two materials over the whole observed temperature range. This reduction temperature leads to an earlier onset of CO production and higher conversion rates at elevated temperatures. This is also reflected by the values reported in Table 1. The maximum activity for NCF-Ni-625 is about 150% of the activities of the other catalysts. Also, *T*_start_ and *T*_middle_ are both reduced by about 40 °C compared to NCF-Ni-550 and NCF-Ni-700. The difference in the H_2_/CO ratio is not very pronounced, but NCF-Ni-625 still exhibits the highest values of H_2_ production at all investigated temperatures. Comparing doped and undoped materials, it becomes even more evident that the reduction at high temperature improves the performance of NCF-Ni-625 the most. All other materials show similar catalytic activity, with the undoped materials even edging out the Ni-doped variants at peak catalytic activity. This fact may be explained by the undoped NCF being more stable than NCF-Ni, which leads to a lower degree of decomposition and deactivation when reduced at 700 °C. For reference, an XRD measurement of a decomposed perovskite (treated at 950 °C in hydrogen) was put into the ESI (Fig. S2).† Additionally, Fig. S15† shows the CH_4_ and CO_2_ conversion values and their respective thermodynamic equilibria. Furthermore, it should be noted that the thermal history of the sample before the reaction reached a maximum of 800 °C during the calcination step. This means that the morphology of the surface is expected to change heavily during the reaction between 800 °C and 950 °C. This leads to the problem of the exact surface area after the reduction being unknown. The surface area changes during the reaction as well. However, it is still necessary to compare the catalysts regarding their surface area. Since only two relatively similar materials were compared at different temperatures, the changes to surface morphology were assumed to be consistent. A surprising result of this work worth noting is the fact that even though the pre-treatments occurred at lower temperatures compared to reaction temperatures, they still influenced the subsequent catalytic performance.

#### 3.1.3. Characterisation after DRM

**Fig. 4:**
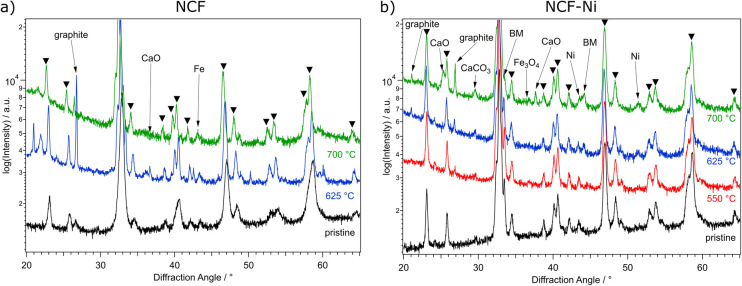
XRD measurements of NCF (a) and NCF-Ni (b) after reduction in H_2_/H_2_O at 500 °C (red, only NCF-Ni), 625 °C (blue), and 700 °C (green), respectively, followed by DRM up to 950 °C as well as the comparison to the pristine catalyst (black). Reflexes marked with triangles can be assigned to the perovskite phase. Reflexes marked by “BM” are assigned to the brownmillerite phase. For NCF, the formation of graphite and CaO as deactivating phases is visible after DRM as well as exsolution of Fe particles. While for the sample that was reduced at 700 °C, CaO was already present after the reduction (Fig. 1a), the phase emerged during DRM for the sample with a lower reduction temperature. NCF-Ni shows the formation of CaO only for the highest reduction temperature. However, the formation of related CaCO_3_ could be observed in all measurements of NCF-Ni. Moreover, Fe and Ni phases were present after the reaction, indicating that the exsolved phases are stable under DRM conditions.

**Fig. 5:**
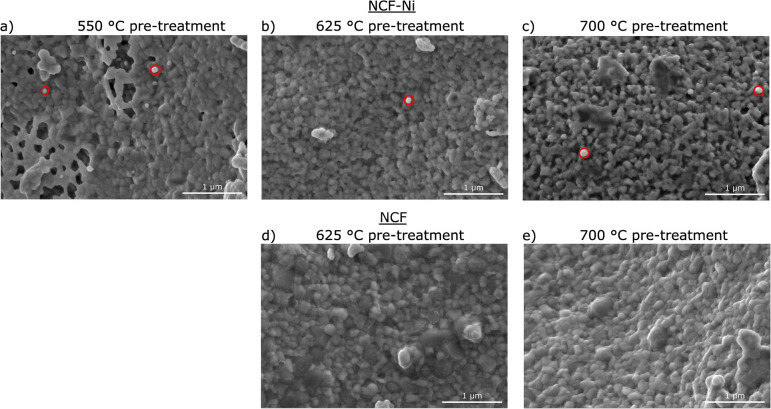
SEM images of NCF (d and e) and NCF-Ni (a–c) after a reductive pre-treatment at 550 °C (a), 625 °C (b and d), and 700 °C (c and e) followed by DRM up to 950 °C, respectively. While for NCF-Ni, some nanoparticles are still visible after the reaction (red circles), the oxide backbone of the catalyst underwent dramatic changes compared to the images after the reduction (Fig. 2).

After DRM, the materials were characterised again using XRD (Fig. 4) and SEM (Fig. 5). Looking at the undoped samples in Fig. 4a, it becomes evident that the catalytic reaction altered the material. At around 26°, a reflex occurred, which can be attributed to the formation of graphite on the catalyst surface. Additionally, CaO was formed. This is a consequence of Ca segregating to the surface and reacting with the DRM atmosphere. Additionally, after reduction at 625 °C, there are a multitude of new reflexes between 36° and 38°, which may be attributed to some iron-oxides forming. All these phases are considered deactivating for the reaction. The Ni-doped catalysts in Fig. 4b show similar phases formed as the undoped material. Additionally, CaCO_3_ was formed after reduction at the highest temperature which happened due to the reaction of Ca from the lattice with CO_2_. The formation of a carbonate phase is a well-known phenomenon during catalytic reactions in the presence of CO_2_.^31,32^ Moreover, metallic Ni could be observed (44.5°) in the measurements even after reduction at 550 °C. This, again, shows the potential for *in situ* exsolution during DRM. Furthermore, it seems that the brownmillerite phase (marked “BM”) that vanished after the reduction emerges again during DRM.

The SEM images in Fig. 5 show a substantial change in the morphology of the catalysts compared to those before the reaction (Fig. 3). For NCF-Ni, spherical nanoparticles are still visible after the reaction, however they have grown to about 150 nm in diameter. However, no nanoparticles could be observed for the undoped materials.

In summary, the materials start to decompose during the reduction at temperatures of 700 °C. However, under a DRM atmosphere, they seem more stable than during the reduction, as only small diffraction peaks of deactivating phases could be observed in the XRD measurements after the reaction (Fig. 4b). However, *in situ* measurements under such conditions are indispensable to understand what is truly happening during these high-temperature reactions.

### 3.2. *In situ* XRD measurements

#### 3.2.1. Measurements during the reduction

**Fig. 6:**
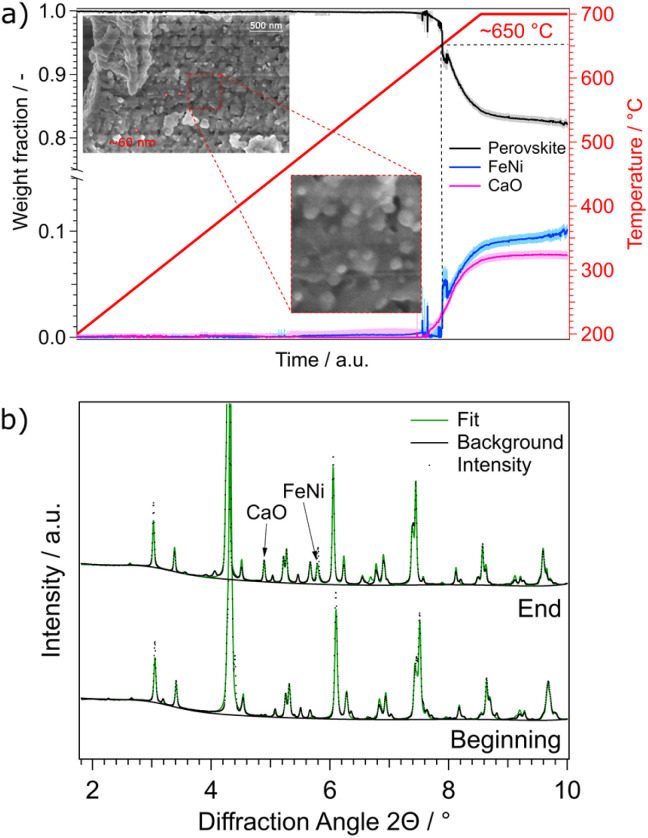
(a) Weight fractions of NCF-Ni during a reduction ramp. The temperature was increased by a rate of 3 °C min^−1^ from 200 °C to 700 °C in H_2_/H_2_O atmosphere. At around 650 °C, a FeNi phase as well as a CaO phase started to form. This indicates the start of the exsolution process as well as the accompanied A-site segregation to compensate for the loss of B-site atoms. SEM images taken after the reduction experiments reveal nanoparticles with a diameter of around 60 nm, consistent with particles observed after reduction at 700 °C in lab-based experiments (Fig. 2c). (b) Comparison between the first and last diffractogram of the reduction. The emerging phases CaO and FeNi are marked.

For more in-depth characterization of the catalysts, a beamtime at DESY Synchrotron Hamburg (beamline P02.1) was conducted for *in situ* XRD with much higher time and spectral resolution. At first, reduction experiments with a temperature ramp (5 °C min^−1^ in humidified hydrogen) were performed to get deeper insights into nanoparticle exsolution and perovskite stability, the results of which are displayed in Fig. 6. Three crystalline phases are forming under these conditions. The perovskite-oxide phase, which is the host material, is accompanied by a metallic FeNi phase and CaO that simultaneously emerge. This follows the expected exsolution process. The temperature is in the expected range (compared to the previous experiments in the lab). In Fig. 6, an onset temperature of around 650 °C is highlighted. It can be seen that both phases emerge at roughly the same temperature, which can be explained by the desire of the perovskite-oxide to keep a balance between A- and B-site occupation. This is referred to as A-site segregation and is currently also a heavily discussed topic in catalysis and electrochemistry research.^33,34^ Interestingly, the amount of FeNi increases even after the initial growth and more metallic phase is forming during the isothermal period at 700 °C. In contrast, the CaO phase does not increase with respect to weight fraction during that time. Comparing the crystallite sizes, which are depicted in Fig. S3b,† the exact opposite behaviour for the weight fractions of the FeNi and CaO phase was observed. Here, the FeNi particles remain the same size (after the initial growth of the crystallites) during the phase of constant temperature, whereas the CaO phase exhibits increasing crystallite size. A possible explanation may be that the exsolved nanoparticles reach their equilibrium size and additional growth is energetically unfavourable compared to the nucleation of additional particles, therefore increasing the weight fraction of the phase without further increases of the crystallite size. In contrast, CaO is forming and continues to grow as crystals using the reservoir of other CaO particles instead of extracting more Ca from the host lattice. This hints towards surface migration, as the CaO particles are not embedded into the surface and, therefore, have a high surface mobility.

**Fig. 7:**
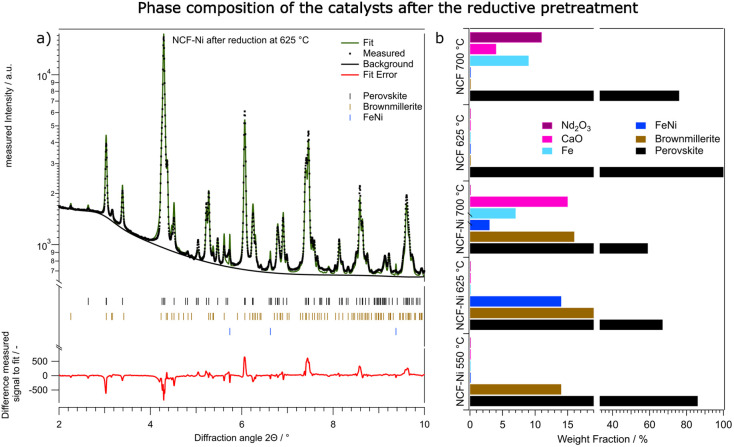
Overview over the phase composition of the catalysts after the reduction at different temperatures. In (a), the fitted diffractogram of NCF-Ni after 1 h in H_2_/H_2_O at 625 °C is shown, including the lines of the phases used for fitting and the difference between the fit and the measured data. (b) shows the qualitative comparison between NCF and NCF-Ni after reduction at the respective investigated temperatures. For both materials, the reduction at 700 °C already led to significant decomposition of the perovskite-oxide host material accompanied by formation of CaO and Nd_2_O_3_. The reduction at 625 °C yielded a stable catalyst consisting of a perovskite phase and an oxygen deficient brownmillerite phase as well as an FeNi phase in case of NCF-Ni. Please note that the *x*-axis on this graph is broken and the area between 20% and 35% is omitted for clarity.

In addition to the experiment with the temperature ramp, the reductive pre-treatments performed in the already mentioned lab-based experiments were repeated. They consisted of one hour of isothermal reductions in humidified hydrogen. In Fig. 7a the fitted diffractogram for NCF-Ni after the reduction at 625 °C is highlighted. Additional features are the positions of the phases and the error of the fit. The two main phases observed after the prolonged reduction are the perovskite backbone of the material as well as a brownmillerite phase, which is related to the perovskite structure but includes an abundance of oxygen vacancies.^35^ The relationship between the perovskite and brownmillerite phases, as well as their transition, have already been explored extensively in literature.^36,37^ Also, metallic FeNi was observed. When looking at the fit error, it becomes clear that all reflexes could be assigned and that the errors are stemming from the intensity of the measured signal. A more-detailed look into the detailed phase evaluation in Fig. S7† reveals that the exsolution of the metallic phases starts about 100 seconds after the pre-treatment – which is consistent with previous work by Neagu *et al.*^15^ After the initial fast exsolution, during which the weight fraction increases abruptly, slower growth happens before the phases remain constant.

Fig. 7b and Table 2 both summarise the composition of all investigated materials and temperatures. While for NCF-Ni, only perovskite and brownmillerite phase are visible at the lowest reduction temperature, increasing the temperature to 625 °C causes the exsolution of a metallic FeNi phase as already discussed. However, a further temperature increase to 700 °C leads to decomposition of the material in form of increased CaO formation. Moreover, a metallic Fe phase could be detected. As all these phases are present as soon as the reduction starts, it can be assumed that the respective kinetics at these temperatures are quite fast. The formation of metallic Fe can be attributed to the limited Ni reservoir in the material or the decomposition of the brownmillerite phase. For the reference material, NCF at 625 °C reduction temperature, only the perovskite phase could be detected. However, increasing the temperature to 700 °C leads to severe decomposition of the material and the formation of Fe followed by Nd_2_O_3_ and CaO. Interestingly enough, the composition mainly changes after around 40 minutes of reduction. This would indicate slower kinetics of the decomposition compared to the exsolution. However, the main findings of the synchrotron-based reductions are consistent with the lab-based experiments, allowing for a qualitative comparison of the effects the reduction has on DRM in the next chapter.

**Table 2:** Overview of the different phase compositions after reduction for one hour at different temperatures. The values are given as weight fractions

	NCF-Ni			NCF	
	550	625	700	625	700
Perovskite	86%	67%	59%	100%	76%
BM	14%	19%	16%	0%	0%
FeNi	0%	14%	3%	0%	0%
Fe	0%	0%	7%	0%	0%
FeO	0%	0%	0%	0%	9%
CaO	0%	0%	15%	0%	4%
Nd_2_O_3_	0%	0%	0%	0%	11%

#### 3.2.2. *In situ* XRD measurements during DRM

**Fig. 8:**
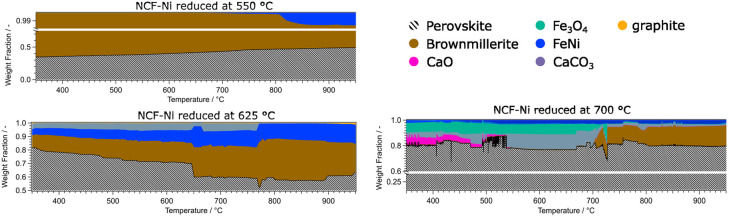
Overview of the phase compositions of the Ni doped catalysts during DRM. It is evident that the perovskite-oxide phase is dominant in all measurements. Additional activating phases (Fe, FeNi) could be observed at reduction temperatures of 625 °C and higher. The formation of graphite was observed at temperatures above 900 °C. At reduction temperatures of 700 °C, deactivating phases such as CaO, CaCO_3_, Fe_3_O_4_ and Nd_2_O_3_ could be detected.

Investigating the phase changes during the DRM reaction reveals deeper insights into the behaviour of the catalysts and can help explain the differences in activity. The calculated weight fractions during the reaction are displayed in Fig. 8 for the Ni doped catalysts and in Fig. S9 (ESI)† for the undoped reference samples. These values result from sequential Rietveld fitting of data gathered during beamtimes at DESY (P02.1, powder XRD beamline). After the reduction discussed in the previous chapter, the materials were cooled down in Ar atmosphere, and the gas phase was subsequently changed to DRM conditions. Afterwards, the temperature was raised by 5 °C min^−1^ to 500 °C and from there by 3 °C min^−1^ to 950 °C. Due to time constraints, the undoped NCF 700 was only heated up to 700 °C under reaction conditions, hence the cut-off in the ESI† figure.

The data for NCF-Ni 550 showed that the material is mainly composed of perovskite and brownmillerite phases. As the temperature rose, the phase ratio shifted to perovskite, meaning that oxygen vacancies were gradually filled during the reaction. Also, at elevated temperatures of around 800 °C, a metallic FeNi phase started to form. This process of *in situ* exsolution under DRM conditions was already investigated in previous work.^22^ In contrast, NCF-Ni 625 already showed a metallic FeNi phase from the beginning, as it was formed during reduction. The amount of the phase continuously grew during the reaction, most likely by *in situ* exsolution and the formation of more particles. Additionally, a CaCO_3_ phase could be detected at lower temperatures. This may be an effect of the change to a CO_2_-containing atmosphere reacting with segregated Ca ions. The amount of CaCO_3_ was constant until about 750 °C. Then, it sharply decreased, and CaO slowly formed (decomposition of CaCO_3_ to CaO). This equilibrium is known to occur at elevated temperatures and can be influenced by transition metals.^38,39^ Additionally, a graphite phase formed during the reaction at temperatures exceeding 900 °C, hinting at slight coke formation on the catalyst surface. The NCF-Ni sample with the highest reduction temperature already showed severe decomposition at the start of the reaction. However, as the reaction increased in temperature, it seemed that the amount of formed CaCO_3_ and Fe_3_O_4_ decreased, and the amount of brown millerite phase increased. A possible explanation would be a solid-state reaction of the decomposed perovskite phase with the decomposition products yielding a brownmillerite phase similar to the calcination process of the synthesis with other precursors.^40^ The undoped material NCF 625 showed similar behaviour to NCF-Ni 550, as the reduction temperature was below the exsolution temperature. However, in contrast to the Ni-doped sample, the reference material showed no initial brownmillerite phase. In this case, metallic Fe was formed during the reaction, presenting another case of *in situ* exsolution. Only at the highest temperatures a brownmillerite phase emerged. The two materials reduced at the highest temperature, NCF-Ni 700 and NCF 700, also revealed interesting parallels: NCF 700 was partially decomposed during the reduction step as well. In contrast to NCF-Ni, however, a brownmillerite phase was present at the beginning of the reaction. The amount of brownmillerite phase increased as decomposition products decreased, mirroring the behaviour observed with the NCF-Ni catalyst. A notable difference was that with NCF 700, the other stable phase was Fe_3_O_4_. However, this measurement was cut short due to time constraints due to the end of the beamtime, so the behaviour at the highest temperature could not be observed.

### 3.3. Discussion

In this work, the effect of a systematic variation of the reduction temperature on a Ni-doped perovskite-oxide catalyst Nd_0.6_Ca_0.4_Fe_0.97_Ni_0.03_O_3_, which was already proven to be a viable DRM catalyst^22^ was shown. This material is still part of fundamental research and not yet viable for industrial applications. Therefore, the A-site still contains stoichiometric amounts of Nd, a critical raw element. However, this work aimed to investigate the effect of different pre-treatment temperatures on the B-site and the formation of nanoparticles. Even though optimisation of the A-site is still necessary, the present work still yields valuable insights into the fundamental processes happening in perovskite-oxides during DRM. These insights might also apply to other CO_2_-converting reactions.

The temperatures were chosen in a way to be below (550 °C), at (625 °C), and above the exsolution temperature of Ni (700 °C), which already leads to the partial decomposition of the catalyst. This was confirmed by *ex situ* XRD analysis of the material after reduction at the respective temperatures. While no additional reflexes were detected at the lowest temperature, compared to the pristine sample, a metallic phase was observed after reduction at 625 °C. A further increase in reduction temperature already led to some decomposition of the material and the formation of CaO phases and metallic Fe, as was observed in previous work with similar materials.^20,23^ As reference, the B-site undoped sample Nd_0.6_Ca_0.4_FeO_3_ was also investigated. For this material, neither a reduction at 550 °C nor 625 °C seemed to have any impact on the material. After treatment at 700 °C, however, a CaO phase formed, seemingly accompanied by traces of metallic Fe. All these findings could also be verified in SEM images taken after the reduction. The doped material showed the formation of spherical nanoparticles after reductions at 625 °C and 700 °C, while the undoped sample showed no such particles. The particles formed during exsolution at 625 °C were slightly smaller than the ones formed at 700 °C (35 nm *vs.* 65 nm), which is in line with literature investigating the size dependence of exsolved nanoparticles in regard to temperature.^16^

Subsequently, the effect that these different reductive pre-treatments have on catalytic performance during DRM was investigated, as it is known the shape and size of the particles can influence the activity.^16^ For each sample, a temperature ramp up to 950 °C was performed in a CH_4_/CO_2_ atmosphere. For the Ni-doped catalyst, it was found that the medium reduction temperature was leading to the highest catalytic reactivity. It was roughly 150% of the ones of the other reduction temperatures. Interestingly – albeit most likely incidentally, the materials prepared at 550 °C and 700 °C exhibited very similar catalytic activity. While after the reduction at 550 °C, no Ni nanoparticles, which are a key reaction site, were present on the surface, the reduction at 700 °C led to the formation of CaO, which covered the active surface area. Another critical aspect, the H_2_/CO product ratio,^1^ was observed as well. Again, the medium reduction temperature of 625 °C performed the best. However, the difference here was not as big as with the catalytic activity. Characterising the materials after the reaction revealed that all materials exhibit some form of degradation during the reaction. XRD measurements showed additional reflexes attributed to CaO, CaCO_3_, graphite and Fe. Furthermore, SEM images revealed that the nanoparticles observed before the reaction were no longer present in case of NCF and exhibited changed morphology for NCF-Ni. All of this indicates that (i) 950 °C is too high for the catalysts to be stable and (ii) the performed *in situ* investigations (see below) were necessary to understand the effect of the reduction temperature on DRM.

*In situ* XRD experiments were performed at DESY during both the reductive pre-treatment and subsequent DRM reactions. A sudden spike of the weight fraction of metallic Ni between 625 °C and 675 °C reduction temperature was observed. After that, the amount of exsolved Ni only slightly increased. However, the crystallite size of the exsolved Ni stayed constant in that regime, indicating that new nanoparticles formed rather than already existing ones grew. A similar effect was observed by Neagu *et al.*, who showed that exsolved nanoparticles reached stability after roughly 100 seconds.^15^ At roughly the same time that Ni was first observed, a CaO phase could also be detected. However, the amount of this phase stayed constant with its crystallite size growing over time. This may indicate that the amount of Ca segregated from the perovskite backbone had already reached equilibrium. However, as the formed CaO is not socketed into the material, its surface mobility remains high, which leads to a subsequent increase in crystallite size. These growth characteristics and kinetics are similar to the ones observed in literature.^18^

Next, the phase changes during isothermal reduction at three different temperatures mentioned previously were investigated. The *in situ* measurements and improved resolution allowed for differentiation and quantification of the perovskite backbone with a secondary brownmillerite phase. These two phases are closely related, but the brownmillerite phase already exhibits a significant amount of ordered oxygen vacancies, which slightly influences the positions of the diffraction reflexes.^41^ For all reduction temperatures, the end state after one hour of reduction was characterised in more detail, as the phase composition did not significantly change in the latter half of the experiment. This leads to the conclusion that thermodynamically speaking, an equilibrium was reached in the reduction experiments. The Ni-doped material resulted in the lowest reduction temperature, showing only an equilibrium between the perovskite and brownmillerite phases. Increasing the reduction temperature to 625 °C gave rise to an additional Ni or mixed FeNi phase. This phase exsolved after around 100 seconds of reduction time and stayed constant for the rest of the reductive pre-treatment. A further increase in reduction temperature again led to decomposition and the formation of deactivating secondary phases such as CaO. For the B-site undoped material, the reduction at 625 °C did not lead to exsolution or decomposition. Only the perovskite phase was detected. Increasing the reduction temperature to 700 °C, already led to significant decomposition as Fe, CaO, and even traces of Nd_2_O_3_ were found.

For the *in situ* DRM experiments the temperature was gradually raised to 950 °C and the respective phases were monitored. The doped variant showed a mix of brownmillerite and perovskite phase for the lowest reduction temperature. At higher temperatures, metallic Ni was exsolved. In previous work, this *in situ* exsolution (without any reductive pre-treatment) was observed at around 600 °C,^22^ consistent with the present study. The Ni-doped catalyst reduced at 625 °C showed surprising behaviour: A CaCO_3_ phase could be observed from the beginning, which was not present at the end of the reduction period. A possible explanation is that the phase formed during the change in atmosphere to CO_2_ and CH_4_, including accelerated Ca segregation. The Ca near the surface reacted with the CO_2_ in the DRM atmosphere, forming the new phase. Combined with the exsolved FeNi phase, the brownmillerite and the perovskite, this phase mixture was stable until around 775 °C. At this temperature, the CaCO_3_ phase started to disappear (CaCO_3_ is decomposing back into CaO), accompanied by a decline in the perovskite phase and an increase in the brownmillerite phase. A possible explanation may be that CaO is partly incorporated into the brownmillerite structure. A trace of CaO remained present in the catalyst even after the mentioned transformation. This re-incorporation was also observed in the samples reduced at 700 °C, which were partly decomposed. FeNi, Fe_3_O_4_, CaO, and CaCO_3_ could be detected for the Ni-doped catalyst next to the perovskite phase. Again, CaCO_3_ was not present after the reduction but could have formed during the transport of the catalyst from the catalytic reactor to the XRD setup. At around 700 °C, the Fe_3_O_4_ phase and CaCO_3_ both disappeared and a new brownmillerite phase formed. The undoped reference material (reduction at 550 °C) remained a mix of brownmillerite and perovskite phase. At temperatures exceeding 700 °C, a metallic Fe phase could be observed that was continuously growing. This *in situ* solution was observed in previous work and literature.^12,22^

**Fig. 9:**
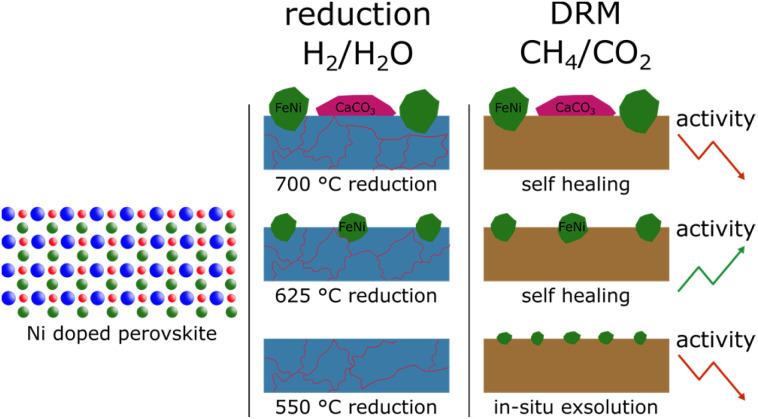
Summary of the main findings. Differently sized FeNi nanoparticles were created from Ni-doped perovskites by utilising different reduction temperatures. At the highest temperature, additional formation of CaCO_3_ could be observed that, in turn, reduced the catalytic activity. During the reduction, the perovskite started to decompose, but under DRM conditions, the decomposition phases formed a brownmillerite phase again presenting a self-healing factor.

A general difference between the Ni-doped and undoped systems is the behaviour of the brownmillerite phase and how decomposition products interact with it. For NCF-Ni, which already showed some brownmillerite phase after the synthesis, Fe_3_O_4_ and CaO formed a brownmillerite phase again at higher temperatures under DRM conditions (Fig. 9). Undoped NCF did not form brownmillerite under reducing conditions but directly decomposed into simple oxides.

## 4 Conclusions

The main takeaways of this study can be summarised in three key messages:

• A dependency of nanoparticle size and formation of secondary phases on the reduction temperature was observed.

• The catalytically active nanoparticles form at similar temperatures as inactive CaO during the reductive pre-treatment. However, the crystallite size of the FeNi phase remained constant, while CaO crystallites continued to grow with rising temperatures.

• *In situ* XRD measurements revealed that the formed phases (both catalytically active and inactive) are in an equilibrium with each other that seems to be temperature-dependent, as the formation of brownmillerite phase from decomposition products of the perovskite (*e.g.*: Fe-oxides, CaCO_3_) was observed.

In the following, these messages are discussed in more detail.

The size of the exsolved nanoparticles and improved the catalytic activity of the material was changed successfully. The known exsolution temperature of 625 °C was increased and decreased by 75 °C, respectively. This led to the formation of nanoparticles for the two higher temperatures, as verified with the SEM images and XRD measurements after the reduction period. This, in turn, led to different catalytic activities. As expected, the lowest reduction period did exhibit low catalytic activity due to the lack of nanoparticles present on the surface. Surprisingly, however, the sample treated to the highest temperature also showed reduced catalytic activity. This was explained by the formation of deactivating phases on the surface, such as CaO.

*In situ* XRD measurements during a temperature ramp under reducing conditions revealed that the metallic phase forming the nanoparticles and the deactivating CaO phase appear at similar temperatures. However, after the initial formation, the amount of the CaO phase remained constant, while its crystallite size continued to grow. In contrast, the metallic FeNi phase exhibited a stable crystallite size with the amount of the phase increasing. This corroborates the known sintering resistance of exsolved nanoparticles in contrast to phases sitting loosely on the surface.

XRD measurements during catalytic measurements revealed that the formed phases are in a constant dynamic equilibrium with each other. For example, CaO and CaCO_3_ are transformed into each other depending on the temperature. Also, the formation of a brownmillerite phase from a partially decomposed perovskite (*e.g.*: Fe-oxides, CaCO_3_) was observed at temperatures above 800 °C. This underscores the stability of perovskite-oxides as catalysts and highlights their potential application in large-scale industrial processes. An observation worth highlighting is that this work showed that while the pre-treatment temperature is lower than the reaction temperature (at most 700 °C *vs.* 950 °C during reaction), the pre-treatment still has significant influence on the catalytic activity. This is worth noting, as a prevalent view in the catalytic community is that the pre-treatments should always be performed at higher temperatures than the reaction to mitigate changes to the surface during the reaction.

In summary, this study highlights the potential applicability of perovskite-oxides for DRM. Even though DRM currently is not implemented in large scale, the fundamental understanding of potential catalytic systems provides valuable insight that can also be transferred to other systems. However, further research, especially into the A-site, is necessary to fully understand the applicability of perovskite oxides for CO_2_-converting reactions.

## Data availability

The data supporting this article have been included as part of the ESI.†

## Author contributions

Conceptualization: C. R., F. S.; formal analysis: F. S., L. L., T. B., funding acquisition: K. F.; C. R; investigation: F. S., T. B., H. D., L. L., R. R.; methodology: F. S.; project administration: C. R.; resources: K. F., C. R.; supervision: C. R.; visualization: F. S.; writing – original draft: F. S; writing – review & editing: T. R.; C. R.

## Conflicts of interest

The authors declare no conflict of interest.

## Supplementary Material

SU-002-D4SU00483C-s001
