# Supplementary material for: How reduction temperature influences the structure of perovskite-oxide catalysts during the dry reforming of methane
Source: RSC Sustain. 2024 Oct 10;2(11):3334–44. doi: 10.1039/d4su00483c (PMC11465801; doi:10.1039/d4su00483c)
Supplement: SU-002-D4SU00483C-s001 [file SU-002-D4SU00483C-s001.pdf]

Supplementary Information (SI) for RSC Sustainability.
This journal is © The Royal Society of Chemistry 2024

How reduction temperature influences the structure of perovskite-oxide catalysts during the dry reforming of methane

Florian Schrenk[a], Lorenz Lindenthal[a], Hedda Drexler[a], Tobias Berger[a], Raffael Rameshan[a], Thomas Ruh[a], Karin Föttinger[b], Christoph Rameshan[a]*

[a]Chair of Physical Chemistry, Montanuniversitaet Leoben, Austria
[b]Institute of Materials Chemistry, TU Wien, Austria

Supporting Info

Pristine catalysts

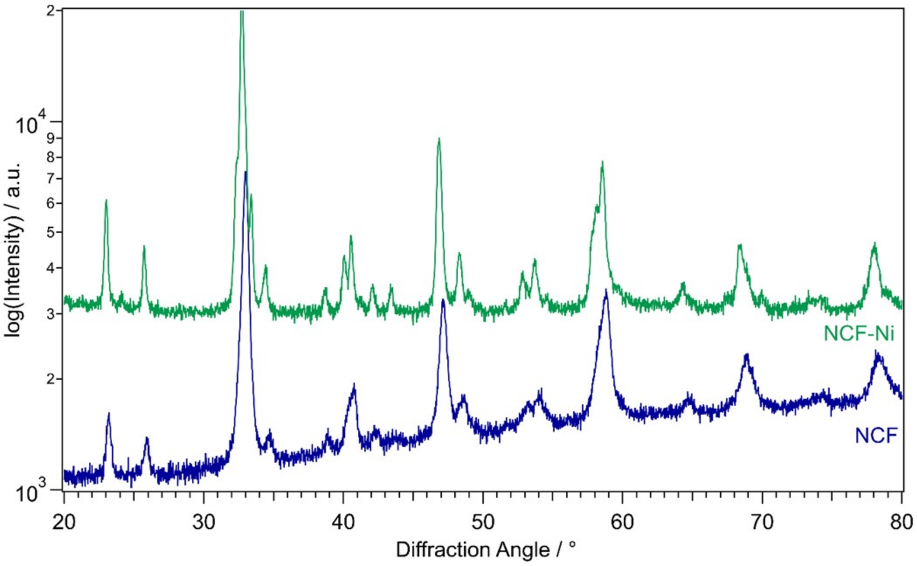

Figure S1: Comparison of the pristine catalyst materials NCF (bottom, blue) and NCF-Ni (green, top)

Table S1: BET surface areas for NCF and NCF-Ni used in the catalytic experiments

Material	BET surface area ($m^2\ g^{-1}$)
NCF	3.78
NCF-Ni	2.43

The pristine catalysts were characterized using XRD to assess phase purity and BET to determine the surface area. The XRD diffractograms show that both materials were successfully synthesized and present in phase pure form. The BET surface area used for calculating the specific activity is in a comparable range, with NCF having a slightly higher surface area than NCF-Ni.

XRD measurement of a decomposed catalyst

To compare the measured catalysts to a decomposed one, the two materials were treated up to 950 °C in a hydrogen-rich atmosphere. CaCO3, various Fe-oxide, and a FeNi phase formed under these conditions.

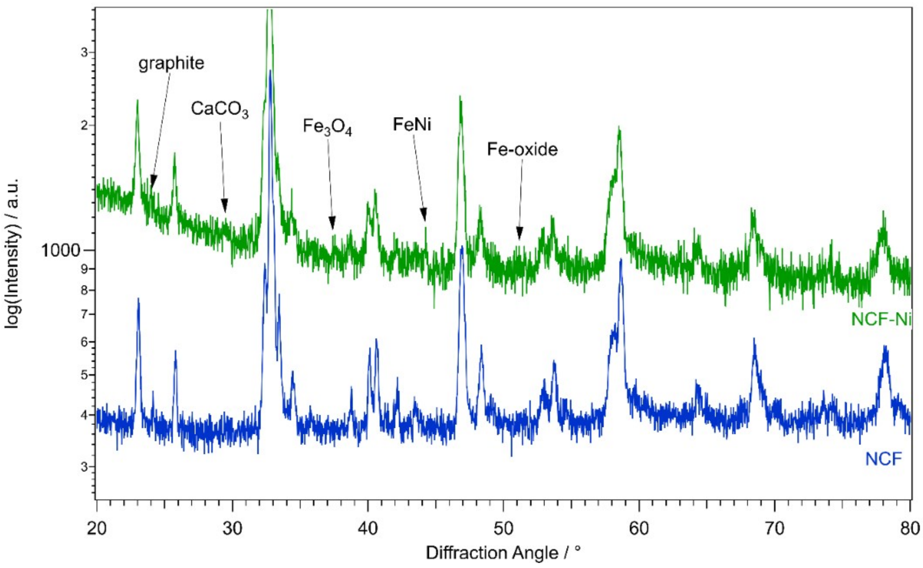

Figure S2: Comparison of the XRD measurements of NCF (blue, bottom) and NCF-Ni (green, top) treated up to 950 °C in a hydrogen-rich atmosphere. Peaks formed after decomposition are labeled. Unlabeled peaks can be assigned to the perovskite structure.

Reductive temperature ramps at DESY

At DESY synchrotron, a temperature ramp in humidified hydrogen (the ratio H_2:H_2O was roughly 32:1) was performed with a heating rate of 3 °C min^{-1} from 200 °C to 700 °C. At 700 °C, the temperature was fixed for an hour. During the temperature program, X-ray diffractograms were acquired continuously with a dwell time of one second. During the data evaluation, 5 diffractograms were pooled into one datapoint leading to a final time resolution of 5 seconds or 0.25 °C during the ramp.

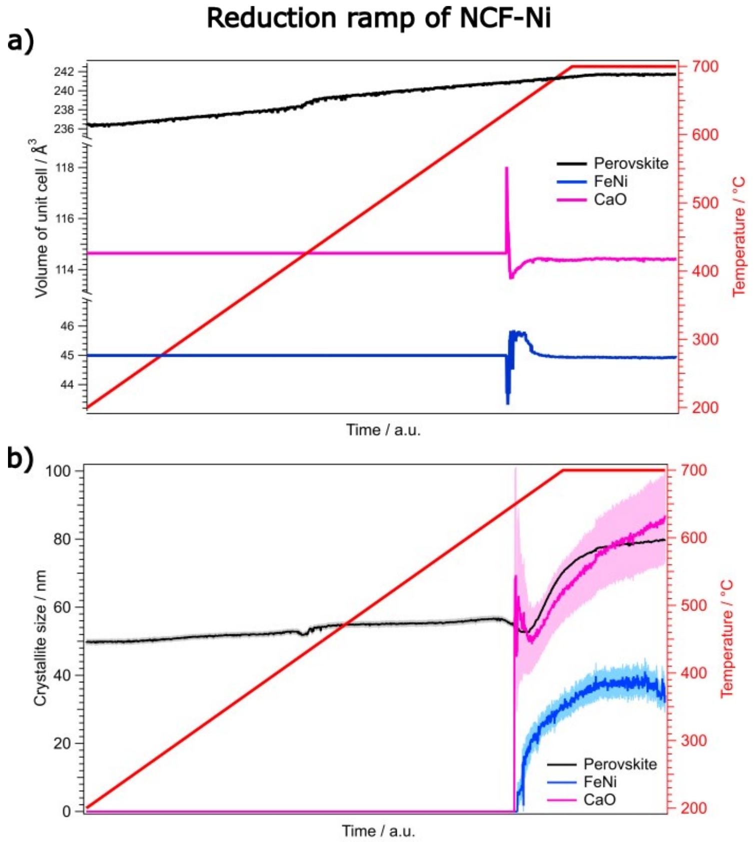

Figure S3: Overview of the volume of the unit cells (a) and crystallite sizes (b) during the reduction ramp of NCF-Ni. The ramp was performed in H_2/H_2O with 3 °C min^{-1} from 200 °C to 700 °C.

As seen in Figure S3, the crystallite sizes of the two emerging phases, FeNi and CaO, can only be calculated once they sufficiently formed at roughly 650 °C. After the initial growth phase, the size of the FeNi crystallites seemed to stabilize at around 35 nm which was in the same size regime as the observed nanoparticles. In contrast, the CaO phase showed continuous growth also during the isothermal phase. In Figure S3a, the unit cell volumes of the observed phases are depicted. Please note that before CaO and FeNi emerged, the nominal unit size is depicted.

Reductive isothermal experiments at DESY

Isothermal reduction experiments at three temperatures (575 °C, 625 °C, and 700 °C) were performed to better compare to the lab-based experiments. Therefore, the sample was brought to the respective temperature in inert Ar flow, before switching to humidified hydrogen for the reduction period for one hour. Data acquisition and treatment were performed as described above.

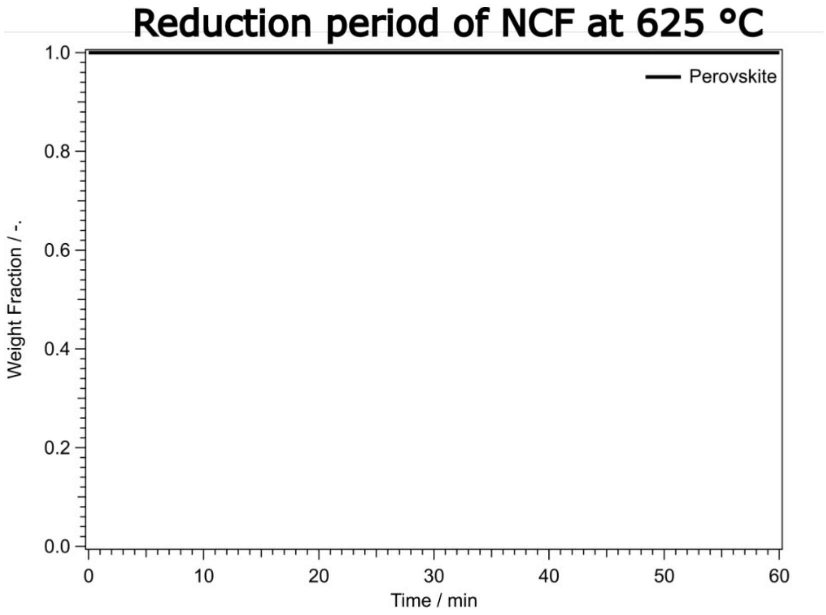

Figure S4: Overview over the weight fraction for the reduction of NCF at 625 °C. The reduction was performed in H_2/H_2O for one hour.

Figure S4 depicts the isothermal reduction period of NCF at 625 °C for one hour. Only the perovskite phase could be detected.

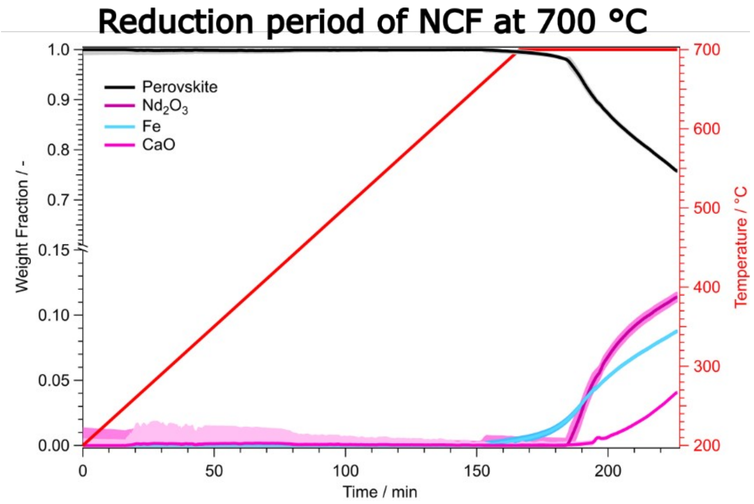

Figure S5: Overview of the weight fraction for the reduction of NCF at 700 °C. The reduction was performed in H_2/H_2O for one hour.

In Figure S5, the results of the reduction of NCF at 700 °C are shown. Because of time constraints at the synchrotron, the NCF reduction at 700 °C was combined with another experiment, including a temperature ramp beforehand. The emergence of a Fe phase was observed close to 700 °C. After that, during the isothermal stage, CaO and Nd_2O_3 emerge.

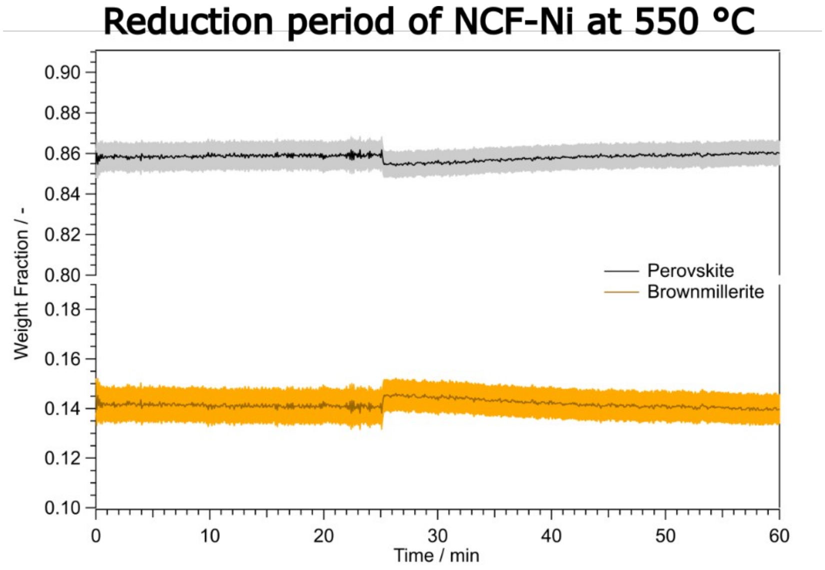

Figure S6: Overview of the weight fractions for the reduction of NCF-Ni at 550 °C. The reduction was performed in H_2/H_2O for one hour.

Figure S6 shows the reduction period of NCF-Ni at 550 °C. For the Ni doped catalyst, a brownmillerite phase formed during the reduction phase. The amounts remained nearly constant throughout the whole duration of the experiment.

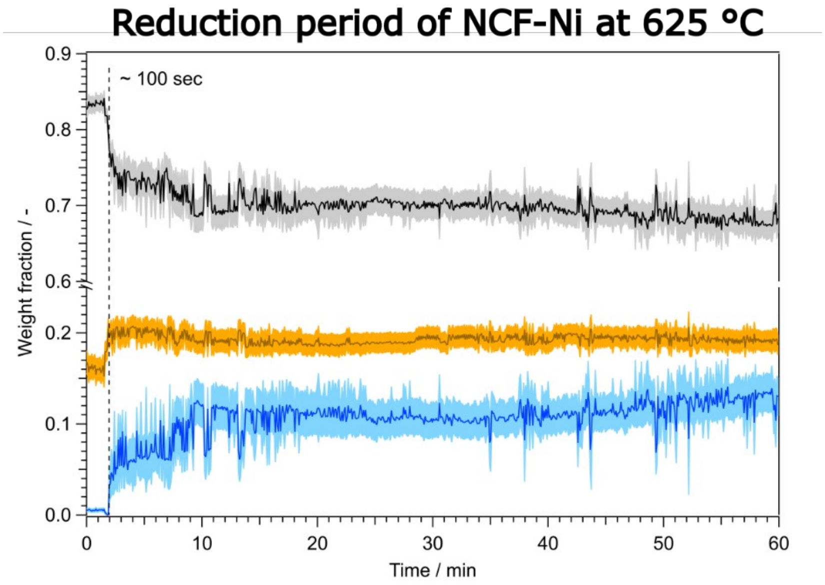

Figure S7: Overview of the weight fractions for the reduction of NCF-Ni at 625 °C. The reduction was performed in H_2/H_2O for one hour.

In Figure S7, the reduction period of NCF-Ni at 625 °C is shown. In the beginning of the reduction, a perovskite phase and a brownmillerite phase were present. After roughly 100 seconds, a metallic FeNi phase emerged, which can be attributed to exsolution. This led to a shift of the crystallite size, mainly for the brownmillerite phase, and a decrease in the weight fraction of the perovskite phase. After that initial change, the phases seemed to stay in equilibrium with each other.

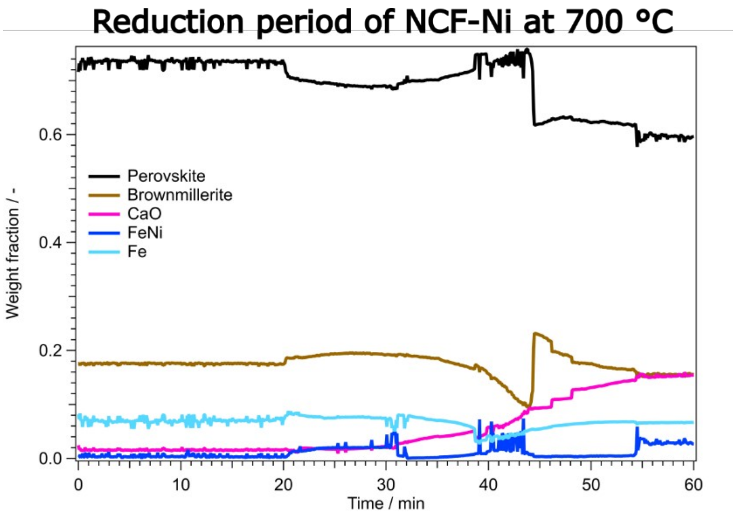

Figure S8: Overview of the weight fraction for the reduction of NCF-Ni at 700 °C. The reduction was performed in H_2/H_2O for one hour.

Figure S8 depicts the reduction period of NCF-Ni at 700 °C. At the beginning of the reduction period, numerous phases were present, including the brownmillerite and perovskite phases as well as a Fe phase that may have formed during heating. During the isothermal process, a FeNi phase and a CaO phase were forming.

Catalytic MDR experiments at DESY

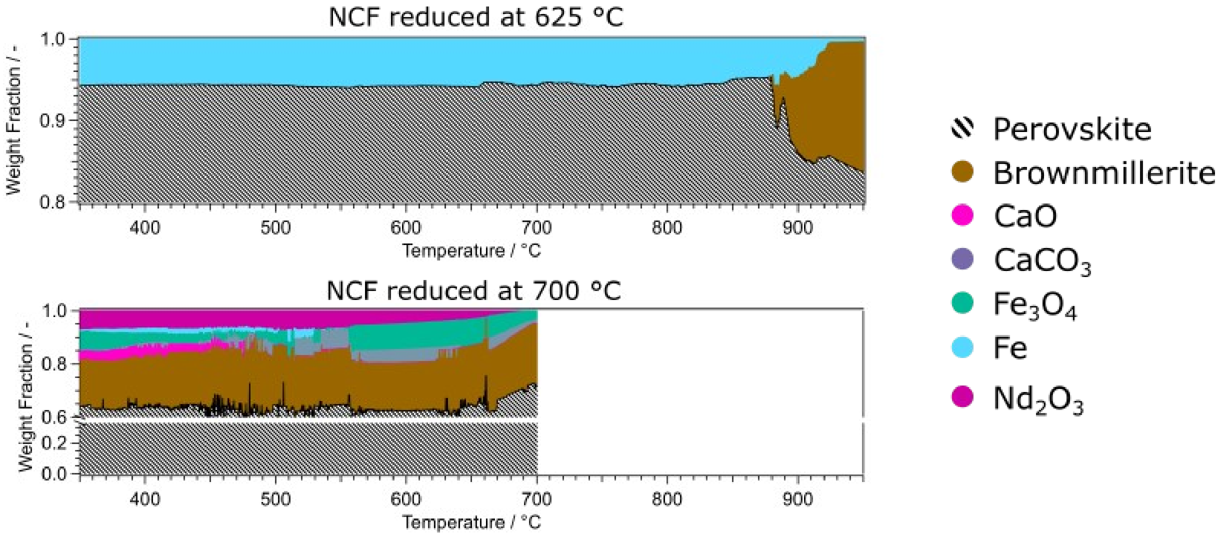

Figure S9: Overview of the phase evolution of the NCF material during MDR after reductions at 625 °C (top) and 700 °C (bottom)

After each isothermal reduction period, the sample was cooled back down to 350 °C in a carefully controlled inert Ar atmosphere. Then, the gases were switched to MDR atmosphere (CH_4:CO_2:Ar = 2:1:3), and a temperature program was performed: The temperature was raised from 350 °C to 500 °C with a rate of 5 °C min^{-1} and then further to 950 °C with a rate of 3 °C min^{-1}. During the whole reaction, XRD data was generated, which was acquired and processed in the same way as described above. Figure S9 gives an overview of the phase evolutions for the undoped catalysts and is discussed in detail in the main manuscript.

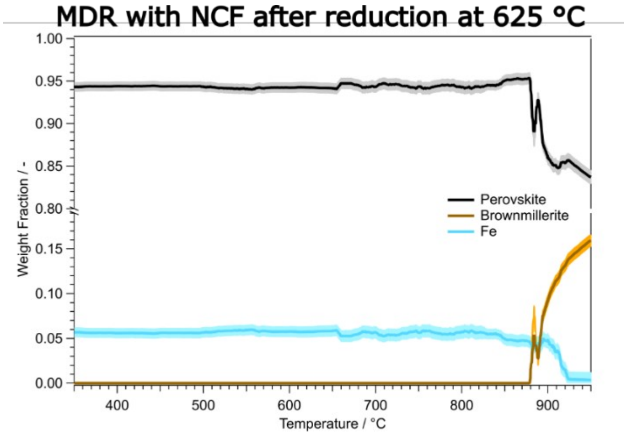

Figure S10: Overview of the weight fractions for DRM with NCF after a reduction at 625 °C. The reaction was performed in a CH_4:CO_2:Ar = 2:1:3 atmosphere with a temperature ramp of 5 °C min^{-1} from 350 °C to 500 °C followed by a ramp of 3 °C min^{-1} from 500 °C to 950 °C.

Figure S10 shows the MDR experiment with NCF after a reduction at 625 °C. At lower temperatures, perovskite and Fe were the sole phases present. At around 880 °C, a brownmillerite phase formed. Please note the broken axis.

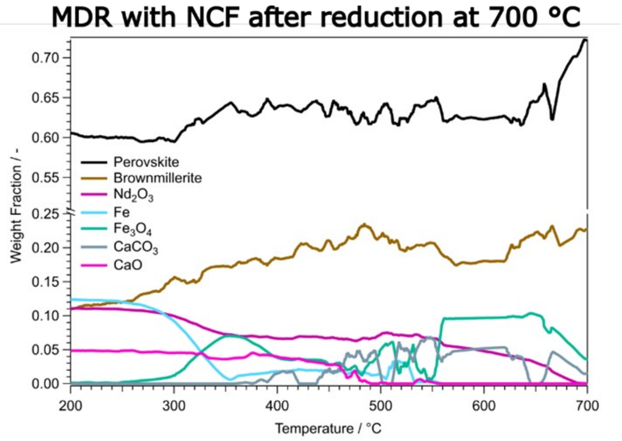

Figure S11: Overview of the weight fractions for DRM with NCF after a reduction at 700 °C. The reaction was performed in a CH_4:CO_2:Ar = 2:1:3 atmosphere with a temperature ramp of 3 °C min^{-1} from 200 °C to 700 °C.

In Figure S11, the weight fractions for the MDR experiment with NCF after reduction at 700 °C are depicted. Please note the broken y-axis. Even though a lot of phases are present, an equilibrium of Fe and Fe_3O_4 can be seen with the oxide forming at higher temperatures and the brownmillerite phase rising with increasing temperature.

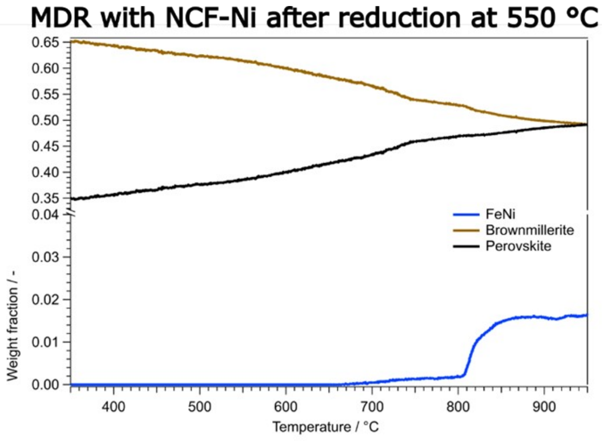

Figure S12: Overview of the weight fractions for DRM with NCF-Ni after a reduction at 550 °C. The reaction was performed in a CH_4:CO_2:Ar = 2:1:3 atmosphere with a temperature ramp of 5 °C min^{-1} from 350 °C to 500 °C followed by a ramp of 3 °C min^{-1} from 500 °C to 950 °C.

Figure S12 depicts the results of the MDR experiment with NCF-Ni after reduction at 550 °C. Initially, only the perovskite and brownmillerite phases were present, but at around 700 °C, a FeNi phase started to form. The weight fraction remains constant until the end of the experiment.

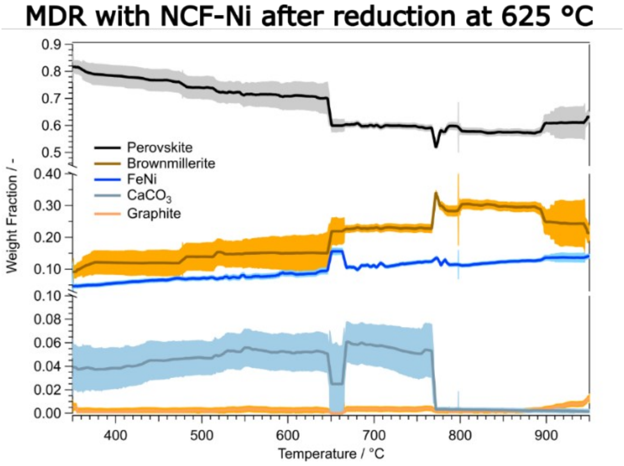

Figure S13: Overview of the weight fractions for DRM with NCF-Ni after a reduction at 625 °C. The reaction was performed in a CH_4:CO_2:Ar = 2:1:3 atmosphere with a temperature ramp of 5 °C min^{-1} from 350 °C to 500 °C followed by a ramp of 3 °C min^{-1} from 500 °C to 950 °C.

In Figure S13, the MDR reaction with NCF-Ni after reduction at 625 °C is depicted. Here, the initially present perovskite, brownmillerite, and FeNi phases seemed to be stable, in contrast to the $CaCO_3$ phase formed in the beginning, which seemed to incorporate into the brownmillerite phase. At temperatures exceeding 900 °C a graphite phase is forming. Please note the twice broken y-axis.

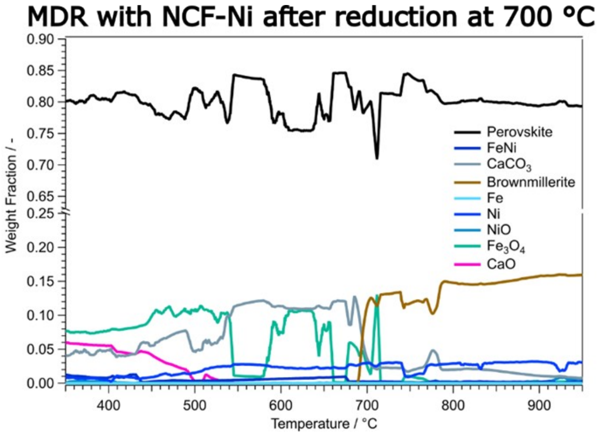

Figure S14: Overview of the weight fractions for DRM with NCF-Ni after a reduction at 700 °C. The reaction was performed in a CH_4:CO_2:Ar = 2:1:3 atmosphere with a temperature ramp of 5 °C min^{-1} from 350 °C to 500 °C followed by a ramp of 3 °C min^{-1} from 500 °C to 950 °C.

Figure S14 shows the results of the MDR experiments of NCF-Ni after reduction at 700 °C. Similar to the undoped counterpart a multitude of phases could be observed. Notable equilibria and transformations include the shift from CaO to $CaCO_3$ with increasing temperature and the increase of the brownmillerite phase while $CaCO_3$ and Fe_3O_4 phases were vanishing. The active FeNi phase seemed to be stable. The sudden spike in FeNi weight fraction at around 350 °C is most likely an experimental artifact, because the catalyst moved in the setup leading to a shift in the diffractogram which the utilized sequential fit misinterpreted.

Catalytic tests

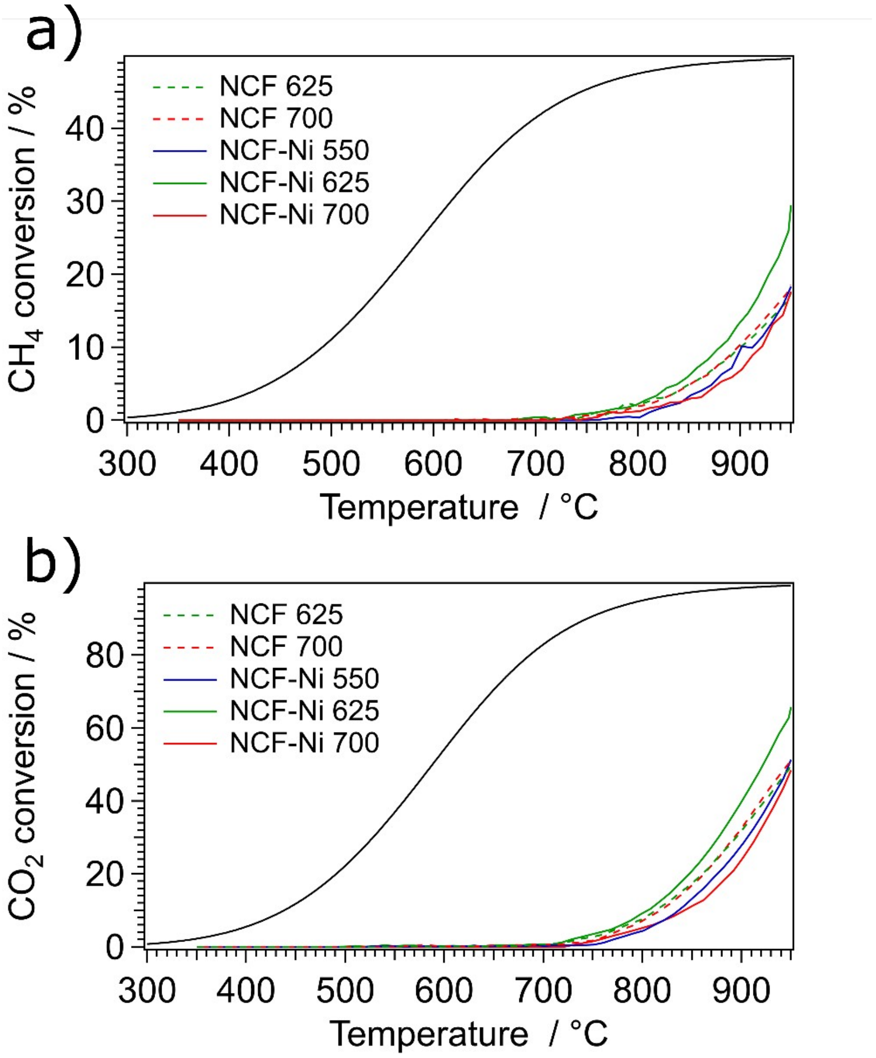

Figure S15: Conversion for CH_4 (a) and CO_2 (b) for the catalytic tests performed in the lab as well as the thermodynamic equilibrium (black line).

Figure S15 shows the conversion of the reactants as well as the calculated thermodynamic equilibrium. None of the reactions came close to the limit, so thermodynamic effects can be excluded.
